# Low-dose aspirin and risk of breast cancer: a Norwegian population-based cohort study of one million women

**DOI:** 10.1007/s10654-023-00976-8

**Published:** 2023-03-06

**Authors:** Lukas Løfling, Nathalie C. Støer, Sara Nafisi, Giske Ursin, Solveig Hofvind, Edoardo Botteri

**Affiliations:** 1 Department of Research, Cancer Registry of Norway, Postboks 5313 Majorstuen, 0304 Oslo, Norway; 2 Norwegian Research Centre for Women’s Health, Women’s Clinic, Oslo University Hospital, Oslo, Norway; 3 Oslo Centre for Biostatistics and Epidemiology, Department of Biostatistics, Institute of Basic Medical Sciences, University of Oslo, Oslo, Norway; 4 Cancer Registry of Norway, Oslo, Norway; 5 Department of Nutrition, Institute of Basic Medical Sciences, University of Oslo, Oslo, Norway; 6 Department of Preventive Medicine, University of Southern California, Los Angeles, USA; 7 Section for Breast Cancer Screening, Cancer Registry of Norway, Oslo, Norway; 8 Department of Health and Care Sciences, UiT The Arctic University of Norway, Tromsø, Norway; 9 Section for Colorectal Cancer Screening, Cancer Registry of Norway, Postboks 5313 Majorstuen, 0304 Oslo, Norway

**Keywords:** Breast cancer, Aspirin, Incidence, Cohort, Nested case–control, Population-based

## Abstract

Several studies evaluated the association between aspirin use and risk of breast cancer (BC), with inconsistent results. We identified women aged ≥ 50 years residing in Norway between 2004 and 2018, and linked data from nationwide registries; including the Cancer Registry of Norway, the Norwegian Prescription Database, and national health surveys. We used Cox regression models to estimate the association between low-dose aspirin use and BC risk, overall and by BC characteristics, women’s age and body mass index (BMI), adjusting for sociodemographic factors and use of other medications. We included 1,083,629 women. During a median follow-up of 11.6 years, 257,442 (24%) women used aspirin, and 29,533 (3%) BCs occurred. For current use of aspirin, compared to never use, we found an indication of a reduced risk of oestrogen receptor-positive (ER +) BC (hazard ratio [HR] = 0.96, 95% confidence interval [CI]: 0.92–1.00), but not ER-negative BC (HR = 1.01, 95%CI: 0.90–1.13). The association with ER + BC was only found in women aged ≥ 65 years (HR = 0.95, 95%CI: 0.90–0.99), and became stronger as the duration of use increased (use of ≥ 4 years HR = 0.91, 95%CI: 0.85–0.98). BMI was available for 450,080 (42%) women. Current use of aspirin was associated with a reduced risk of ER + BC in women with BMI ≥ 25 (HR = 0.91, 95%CI: 0.83–0.99; HR = 0.86, 95%CI: 0.75–0.97 for use of ≥ 4 years), but not in women with BMI < 25.Use of low-dose aspirin was associated with reduced risk of ER + BC, in particular in women aged ≥ 65 years and overweight women.

**Supplementary Information:**

The online version contains supplementary material available at 10.1007/s10654-023-00976-8.

## Introduction

Breast cancer (BC) is the most diagnosed cancer and the leading cancer-related cause of death in women worldwide [1]. In 2020, it was estimated that approximately 2.3 million new cases of BC were diagnosed, and 700,000 women died of BC. Since the 1980s, the incidence of BC has increased in many high-income countries. Recently, the incidence of BC has also started to increase in many low- and middle-income countries, mainly due to changes in lifestyle factors, such as increased body mass index (BMI), physical inactivity, and postponement of childbearing.

Aspirin, mainly in low doses, is routinely used for the prevention of cardiovascular diseases, such as heart attack and stroke. Use of aspirin has been associated with a reduced risk of different types of cancer, in particular colorectal cancer [2]. Several epidemiological studies have assessed the association between use of aspirin and the risk of BC, with inconsistent results [3]. The exact mechanism for the anti-cancer effect of aspirin remains unclear. One of the main hypotheses is related to the inhibition of cyclooxygenase (COX) enzymes, which convert arachidonic acid into prostaglandins [4]. Prostaglandins play a role in cancer development as they suppress apoptosis, and promote cell proliferation, invasiveness, and migration. Furthermore, decreased levels of prostaglandins may also result in lower levels of oestrogens by downregulating aromatase, an enzyme involved in converting androgen to oestrogen in peripheral fat tissue, and consequently a reduction in the risk of oestrogen receptor-positive (ER +) BC [5, 6].

Previous studies on the association between aspirin and risk of BC have often been impaired by the small size of the study population, use of self-reported data on aspirin use, and the lack of information on tumours’ and women’s characteristics, which have made it difficult to examine possible associations in depth. In this study, we used a large nationwide population-based cohort consisting of more than 1 million women, and we investigated the association between use of low-dose aspirin and the risk of BC according to tumours’ characteristics, such as stage or molecular subtype, and women’s characteristics, such as age and BMI.

## Methods

### Data sources and study population

All Norwegian residents are assigned an 11-digit unique personal identification number at birth or immigration. The personal identification number is included in all national registries and allows for linkage between them. To explore the influence of low-dose aspirin on the risk of BC, we linked individual-level data from different population-based registries. Data on dispensed prescriptions were provided by the Norwegian Prescription Database, which collects detailed information on all dispensed prescriptions from community pharmacies on an individual level since January 2004 [7]. The database includes information on, for example, the date of dispensation, Anatomical Therapeutic Chemical (ATC) code for the dispensed medications, amount dispensed (i.e., number of units dispensed, e.g., number of tablets), strength (i.e., amount of active pharmaceutical ingredient per unit, e.g., mg per tablet), and defined daily doses (DDD). DDD is defined as the average maintenance dose per day for a medication used for its main indication in adults [8]. Data on cancer diagnoses were provided by the Cancer Registry of Norway [9]. The Cancer Registry of Norway started recording incident cancer cases in 1953. For the registration period 2001–2005, the overall completeness of the Cancer Registry of Norway was estimated at 99%, with 99% of the BC cases being histologically verified [10]. Statistics Norway provided information on the date of birth, migration, educational level, income, marital status, country of origin, and the number of children [11]. Information on the date of death and cause of death were provided by the Cause of Death Registry [12]. Information on BMI was obtained from a subset of women who had participated in different health surveys (The Cohort of Norway [CONOR] [13], Romsås in Motion [MoRo1] [14]), and the Norwegian mammography database [15]. We used the measurement closest in time to the start of follow-up, with a maximum of 5 years before or after the start of follow-up.

Using the Population Registry from Statistics Norway [11], we identified all women born in 1925–1986 who lived in Norway at any time between 1st January 2004 and 31st December 2018. We followed all women who lived in Norway for at least 6 months after the cohort entry date (1st January 2004, the first immigration, or the date they turned 49.5 years, whichever occurred last). Follow-up started 6 months after the cohort entry date. We included only women aged ≥ 50 years at the start of follow-up for the following reasons. Low-dose aspirin use in the prevention of cardiovascular diseases and colorectal cancer has usually been recommended to individuals aged ≥ 50 years [16]. Therefore, we consider women aged ≥ 50 years to be the relevant population from a clinical practice point of view and a public health point of view. Moreover, the restriction to women aged ≥ 50 years was planned also to have a more homogeneous study population, which includes mostly post-menopausal women from the age they are invited to the mammography screening [15]. Finally, the fact that use of low-dose aspirin is rare among women aged < 50 years would have resulted in unstable estimates among these women, especially when stratified by women’s or BC’s characteristics. We excluded women with a known history of invasive cancer before the start of follow-up (except invasive non-melanoma skin cancer [International Classification of Diseases [ICD] version 10 code: C44]). Women were followed-up until a diagnosis of BC (outcome of interest), a diagnosis of another form of cancer, death, emigration, or administrative censoring (31^st^ December 2018), whichever occurred first.

### Exposure assessment

Use of low-dose aspirin (ATC code: B01AC06 and B01AC56) was based on dispensed prescriptions recorded in the Norwegian Prescription Database. We did not have access to data on dispensed prescriptions of aspirin in regular-dose (ATC code: N02BA01) used as analgesic. The total number of treatment days was calculated using the number of DDDs, assuming 1 DDD per day. The estimated duration of low-dose aspirin was extended by 4 months (grace period) to account for prolonged use beyond the estimated treatment days. Use of low-dose aspirin was handled in a time-varying way, meaning that all women may have contributed person-time at risk as a never-user, current user, and past user. Women who were not dispensed a prescription of low-dose aspirin in the 6 months before the start of follow-up contributed time at risk as a never-user from the start of follow-up until the date of the first possible dispensed prescription of low-dose aspirin (Supplementary Fig. 1). Women contributed person-time at risk as a current user from the date of the dispensed prescription until the end of the estimated duration of low-dose aspirin (i.e., end of the grace period). If there were gaps between the end of the estimated duration and the next possible dispensed prescription of low-dose aspirin, women contributed person-time at risk as a past user from the end of the estimated duration of low-dose aspirin (i.e., end of the grace period) until the next dispensed prescription or end of follow-up. Women with a period of current use covering the date of start of follow-up, started to contribute person-time at risk as a current user from the start of follow-up. Women who were dispensed a prescription of low-dose aspirin in the 6 months before the start of follow-up, with an estimated duration not covering the start of follow-up started to contribute person-time as a past user from the start of follow-up.

### Outcome assessment

A diagnosis of BC (ICD-10: C50, carcinomas only [ICD morphology codes: 801–823, 825–867, 894], i.e., excluding lymphomas, sarcomas, and carcinoids) was the outcome of interest. To categorise the molecular subtypes of BC, we used information from the Cancer Registry of Norway on ER status, progesterone receptor (PR) status, human epidermal growth factor receptor 2 (HER2) status and Ki-67 level [17]; luminal A (ER + and/ or PR + , HER2-, Ki-67 ≤ 14), luminal B HER2- (ER + and/ or PR + , HER2-, Ki-67 > 14), luminal B HER2 + (ER + and/ or PR + , HER2 +), HER2 + (ER-, PR-, HER2 +), and triple-negative BC (TNBC) (ER-, PR-, HER2-). In the case of missing information on Ki-67, we used tumour grade I for luminal A, and II-III for luminal B HER2- [18]. Information on the BC stage in the Cancer Registry of Norway was categorised as local, regional, or distant according to the United States National Cancer Institute’s Surveillance, Epidemiology, and End Results (SEER) Program [19].

### Study design

We applied a cohort design to estimate the association between use of low-dose aspirin and risk of BC. In addition, we applied a nested case–control design to categorise low-dose aspirin use in different intervals of duration (never, past, current use of < 2, 2–3.9 and ≥ 4 years) and to assess a potential trend in the risk of BC by the duration of low-dose aspirin use. The duration of low-dose aspirin use was assessed in the last treatment episode before the index age (i.e., age at BC diagnosis for the cases and the corresponding age for the matched controls). We excluded women who stayed < 4 years in the cohort (i.e., women with < 4 years from start of follow-up until a BC diagnosis or censoring). In this way, we were able to categorise the women to have been a user for ≥ 4 years if the last treatment episode was a period of current use which lasted for ≥ 4 years. The 4-year limit was decided based on a trade-off between not excluding too many women and setting a minimum length (4 years in our study) that could be considered as intermediate/long-term use. The BC cases identified in the cohort study were individually matched with 10 controls from the cohort who were still at risk of BC at the age when the case got the BC diagnosis. In the analyses of the nested case–control study stratified by BMI, the cases and controls were also matched on BMI (< 25 kg/m^2^, ≥ 25 kg/m^2^).

### Statistical analysis

Cox proportional hazard models were used to estimate hazard ratios (HR) with 95% confidence intervals (CI) for the association between use of low dose-aspirin and risk of BC in the cohort design. Attained age was used as the underlying time scale. In the nested case–control design, we used conditional logistic regression models to estimate the HR and 95% CI for the association between the duration of low-dose aspirin use and risk of BC. The *p*-value for trend, in the nested case–control study, was estimated by entering the categorical exposure variable (never, past, current < 2, 2–3.9 and ≥ 4 years) as a continuous variable (values from 0 to 4) in the models.

The Cox proportional hazard models and the conditional logistic regression models were adjusted for a priori selected covariates collected at the start of follow-up in the analysis of the cohort study and at the index age in the analysis of the nested case–control study: age in years, education (none/primary school only, secondary school, university, missing), income quartiles, marital status (married/partnered, not married/partnered, missing) country of origin (Norway, other Nordic countries [i.e., Denmark, Finland, Iceland, Sweden], rest of the world), number of children (0, 1, 2, ≥ 3), and ever use (≥ 1 dispensed prescription) of other anti-platelets, beta-blockers, angiotensin-converting enzyme (ACE)-inhibitors, angiotensin receptor blockers (ARB), calcium channel blockers (CCB), diuretics, statins, antidiabetics, non-steroidal anti-inflammatory drugs (NSAID), and menopausal hormone therapy (HT) (for ATC-codes, see Supplementary Table 1). We adjusted for the different medications for 3 different reasons: 1) because they are possible confounders (i.e., associated with both aspirin use and risk of BC), 2) they are used to treat conditions that are possible confounders (we use them as a proxy for the condition since we do not have information on comorbid conditions), or 3) to control for possible underlying risk factors of those comorbid conditions (e.g., smoking, diet, physical activity, or alcohol consumption). Women were categorised as ever users in the time after the first dispensed prescription in the cohort study and in the period before the index age in the nested case–control study. Missing information on covariates was handled as a separate category in the variable. We performed analyses overall, and separately in subgroups defined by ER status, molecular subtype, stage, women’s age (attained age in the cohort study, and index age in the nested case–control study: 50–64.9 years, 65–94 years), and BMI (< 25 kg/m^2^, ≥ 25 kg/m^2^).

In addition to the main analyses, we performed sensitivity analyses where we assessed the association between use of low-dose aspirin and BC risk separately for screen-detected BC and symptomatic BC (including both interval-detected BC and BC detected outside the screening program), where we censored for the other mode of detection. We also performed an analysis where we censored women at the time they were dispensed a prescription of HT, a well-established risk factor for BC [20]. Finally, in order to assess the influence of the length of the grace period, we performed analyses where we changed the grace period from 4 months to 2 and 6 months.

All tests were two-sided with a 5% significance level. Statistical analyses were performed using R software version 3.4.4 or later (http://cran.r-project.org/).

### Ethics approval

The study received ethics approval from the Regional Committee for Medical and Health Research Ethics Sør-Øst (S-09113b 2009/2062, 2014/1854/REK sør-øst B) and from the Norwegian Data Protection Authority (17/00222–4/GRA). For the registry data and the data from the Norwegian mammography database, informed consent from the included study subjects was not required according to Norwegian law. For the included national health surveys, informed consent was collected from the participants at inclusion.

## Results

We identified 1,872,600 Norwegian women who lived in Norway at any time between 2004 and 2018. We excluded 788,971 women because they lived less than 6 months in Norway (*n* = 15,311), had a history of cancer (*n* = 64,064) and were < 50 years at the end of follow-up (*n* = 709,596). Hence in total, we included 1,083,629 women. During a median follow-up of 11.6 years, 257,442 (24%) women were classified as ever users of low-dose aspirin (83% of the prescriptions were 75 mg aspirin and 17% were 160 mg aspirin) and 29,533 (3%) BCs were diagnosed. At the start of follow-up, the women who never used low-dose aspirin during the study period were younger, more educated, had a higher income, were more likely to be nulliparous, and less often used other medications than the women who used low-dose aspirin (Table 1).

**Table 1:** Baseline characteristics by use of low-dose aspirin, Norway 2004–2018

	No low-dose aspirin (*N* = 826,187)	Low-dose aspirin (*N* = 257,442)
*Age (years) at the start of follow-up*		
Median (Q1, Q3)	50.0 (50.0, 58.8)	62.6 (54.7, 71.3)
*Highest education level*		
None/primary school	211,197 (25.6%)	99,160 (38.5%)
Secondary school	350,244 (42.4%)	114,041 (44.3%)
University	233,135 (28.2%)	41,695 (16.2%)
Missing	31,611 (3.8%)	2546 (1.0%)
*Income (Norwegian kroner)*		
Q1 (< 154,000)	165,160 (20.0%)	100,898 (39.2%)
Q2 (154,000–258,000)	185,197 (22.4%)	80,860 (31.4%)
Q3 (258,001–385,000)	215,196 (26.0%)	50,861 (19.8%)
Q4 (> 385,000)	241,733 (29.3%)	24,324 (9.4%)
Missing	18,901 (2.3%)	499 (0.2%)
*Marital status*		
Married/partnered	315,361 (38.2%)	102,736 (39.9%)
Not married/partnered	487,302 (59.0%)	153,373 (59.6%)
Missing	23,524 (2.8%)	1333 (0.5%)
*Country of origin*		
Norway	712,745 (86.3%)	238,051 (92.5%)
Other Nordic countries^a^	28,987 (3.5%)	5584 (2.2%)
Rest of the world	84,455 (10.2%)	13,807 (5.4%)
*Children*		
0	115,031 (13.9%)	24,828 (9.6%)
1	109,813 (13.3%)	32,398 (12.6%)
2	316,960 (38.4%)	91,920 (35.7%)
≥ 3	284,383 (34.4%)	108,296 (42.1%)
*BMI (kg/ m*^*2*^*)*		
< 25	179,536 (21.7%)	38,346 (14.9%)
≥ 25	176,026 (21.3%)	56,172 (21.8%)
Missing	470,625 (57.0%)	162,924 (63.3%)
*Ever use of other drugs*		
Other anti-platelets	8027 (1.0%)	62,884 (24.4%)
Beta-blockers	124,763 (15.1%)	147,168 (57.2%)
Calcium channel blockers	109,621 (13.3%)	107,050 (41.6%)
Angiotensin-converting enzyme inhibitors	60,371 (7.3%)	70,256 (27.3%)
Angiotensin receptor blockers	161,072 (19.5%)	119,317 (46.3%)
Diuretics	180,535 (21.9%)	150,896 (58.6%)
Statins	156,505 (18.9%)	186,762 (72.5%)
Antidiabetics	43,906 (5.3%)	42,711 (16.6%)
Non-steroidal anti-inflammatory drugs	564,833 (68.4%)	205,613 (79.9%)
Menopausal hormone therapy	305,196 (36.9%)	113,028 (43.9%)
*Breast cancer detection mode*^*b*^		
Screen-detected	7379 (31.3%)	1144 (19.3%)
Symptomatic breast cancer	16,224 (68.7%)	4786 (80.7%)

*Abbreviations* Quartile (Q), Body mass index (BMI)

^a^Includes Denmark, Finland, Iceland, and Sweden

^b^Only including women with a breast cancer diagnosis

In the nested case–control study used for assessing the association between the duration of low-dose aspirin use and BC risk, we included 20,523 cases and 205,230 matched controls. At the start of follow-up, the women included in the nested case–control study were older, had a lower income, and were less often married compared to the women in the full cohort (Supplementary Table 2). At index age, the cases were more educated and had a higher income than the controls (Supplementary Table 3).

BMI was available for 450,080 (42%) women. The median time between the BMI measurement and the start of follow-up was 2 years after the start of follow-up (1st quartile: 1 year after, 3rd quartile: 3 years after). At the start of follow-up, women with a BMI measurement were more educated, had a higher income, and were less often married compared to the women in the full cohort (Supplementary Table 2).

### Association between use of low-dose aspirin and BC incidence

In the total population, HR for the association between current use of low-dose aspirin, as compared to never use, and risk of any type of BC, was 0.98 (95% CI: 0.94–1.02) (Table 2), while the HR for past use was 0.99 (95% CI: 0.94–1.05). The HRs for the association between current use of low-dose aspirin, as compared to never use, and risk of ER + BC and ER- BC were estimated at 0.96 (95% CI: 0.92–1.00) and 1.01 (95% CI: 0.90–1.13), respectively. The association with ER + BC was observed among women aged ≥ 65 years (HR = 0.95, 95% CI: 0.90–0.99), but not in women aged 50–64.9 years (HR = 0.99, 95% CI: 0.92–1.07). The association was mainly driven by luminal A BC (HR = 0.92, 95% CI: 0.85–0.99 in the overall population; HR = 0.90, 95% CI: 0.82–0.99 among women aged ≥ 65 years). For ER + BC, in women aged ≥ 65 years, we found a trend in the association by duration of low-dose aspirin use, HRs for past use and current use of < 2, 2–3.9 and ≥ 4 years were 1.01 (95% CI: 0.94–1.09), 1.02 (95% CI: 0.93–1.12), 0.94 (95% CI: 0.84–1.06) and 0.91 (95% CI: 0.85–0.98), respectively (*p*-value for trend = 0.016) (Table 3). We found no trend in women aged 50–64.9 years. When ER status was simultaneously stratified by cancer stage and age, we found no evidence in support of an association with reduced risk of BC in any of the subgroups (Table 4).

**Table 2:** Association between use of low-dose aspirin and incidence of breast cancer in Norway 2004–2018, by molecular subtype and age

	Low-dose aspirin	Cases	Person-years	HR^a^ (95% CI)	Age (years)					
					50–64.9			≥ 65		
					Cases	Person-years	HR^a^ (95% CI)	Cases	Person-years	HR^a^ (95% CI)
Total population	*Never use*	23,603	8,670,359	1 (ref.)	14,598	5,543,101	1 (ref.)	9005	3,127,258	1 (ref.)
	*Current use*	4183	1,497,420	0.98 (0.94–1.02)	1128	402,125	1.02 (0.95–1.10)	3055	1,095,295	0.96 (0.91–1.01)
	*Past use*	1747	586,836	0.99 (0.94–1.05)	425	161,296	0.91 (0.83–1.01)	1322	425,541	1.02 (0.96–1.08)
*Molecular subtype*										
ER +	*Never use*	18,682	8,670,359	1 (ref.)	11,735	5,543,101	1 (ref.)	6947	3,127,258	1 (ref.)
	*Current use*	3201	1,497,420	0.96 (0.92–1.00)	902	402,125	0.99 (0.92–1.07)	2299	1,095,295	0.95 (0.90–0.99)
	*Past use*	1423	586,836	1.03 (0.97–1.09)	355	161,296	0.92 (0.83–1.03)	1068	425,541	1.05 (0.98–1.13)
ER-	*Never use*	2830	8,670,359	1 (ref.)	1819	5,543,101	1 (ref.)	1011	3,127,258	1 (ref.)
	*Current use*	507	1,497,420	1.01 (0.90–1.13)	135	402,125	0.96 (0.79–1.18)	372	1,095,295	1.02 (0.89–1.17)
	*Past use*	221	586,836	1.08 (0.93–1.25)	57	161,296	0.99 (0.76–1.31)	164	425,541	1.11 (0.93–1.32)
Luminal A	*Never use*	6203	8,670,359	1 (ref.)	3892	5,543,101	1 (ref.)	2311	3,127,258	1 (ref.)
	*Current use*	984	1,497,420	0.92 (0.85–0.99)	307	402,125	0.96 (0.83–1.10)	677	1,095,295	0.90 (0.82–0.99)
	*Past use*	454	586,836	1.01 (0.91–1.12)	123	161,296	0.89 (0.74–1.08)	331	425,541	1.06 (0.93–1.20)
Luminal B HER2-	*Never use*	9122	8,670,359	1 (ref.)	5706	5,543,101	1 (ref.)	3416	3,127,258	1 (ref.)
	*Current use*	1608	1,497,420	0.97 (0.91–1.03)	435	402,125	1.00 (0.89–1.12)	1173	1,095,295	0.96 (0.89–1.04)
	*Past use*	727	586,836	1.06 (0.97–1.15)	182	161,296	0.99 (0.85–1.15)	545	425,541	1.07 (0.97–1.18)
Luminal B HER2 +	*Never use*	1898	8,670,359	1 (ref.)	1276	5,543,101	1 (ref.)	622	3,127,258	1 (ref.)
	*Current use*	288	1,497,420	0.93 (0.80–1.07)	88	402,125	0.96 (0.75–1.23)	200	1,095,295	0.91 (0.76–1.09)
	*Past use*	123	586,836	0.98 (0.81–1.19)	37	161,296	0.97 (0.69–1.36)	86	425,541	0.97 (0.76–1.24)
HER2 +	*Never use*	894	8,670,359	1 (ref.)	603	5,543,101	1 (ref.)	291	3,127,258	1 (ref.)
	*Current use*	144	1,497,420	1.00 (0.81–1.23)	47	402,125	1.01 (0.71–1.43)	97	1,095,295	0.99 (0.76–1.30)
	*Past use*	49	586,836	0.81 (0.60–1.10)	11	161,296	0.58 (0.32–1.07)	38	425,541	0.91 (0.64–1.31)
TNBC	*Never use*	1617	8,670,359	1 (ref.)	1021	5,543,101	1 (ref.)	596	3,127,258	1 (ref.)
	*Current use*	303	1,497,420	0.99 (0.85–1.15)	73	402,125	0.92 (0.70–1.22)	230	1,095,295	1.01 (0.85–1.21)
	*Past use*	149	586,836	1.19 (0.99–1.43)	40	161,296	1.22 (0.88–1.69)	109	425,541	1.18 (0.95–1.47)

*Abbreviations* Hazard ratio (HR), Confidence interval (CI), Oestrogen receptor (ER), Human epidermal growth factor receptor 2 (HER2), Triple-negative breast cancer (TNBC)

^a^Adjusted for age in years (the underlying time scale), education, income quartiles, marital status, country of origin, number of children and ever use of other anti-platelets, beta-blockers, angiotensin-converting enzyme inhibitors, angiotensin receptor blockers, calcium channel blockers, diuretics, statins, antidiabetics, non-steroidal anti-inflammatory drugs, and menopausal hormone therapy

**Table 3:** Association between duration of low-dose aspirin use and incidence of breast cancer in Norway 2004–2018, by molecular subtype and age (*n* = 225,753)

	Low-dose aspirin	Cases	Controls	HR^a^ (95% CI)	Age at index (years)					
					50–64.9			≥ 65		
					Cases	Controls	HR^a^ (95% CI)	Cases	Controls	HR^a^ (95% CI)
Total population	*Never use*	15,570	155,866	1 (ref.)	8166	81,617	1 (ref.)	7404	74,249	1 (ref.)
	*Past use*	1621	15,500	0.99 (0.94–1.05)	366	3845	0.93 (0.83–1.05)	1255	11,655	1.01 (0.94–1.08)
	*Use* < *2 years*	983	9543	1.02 (0.95–1.09)	265	2690	1.00 (0.87–1.14)	718	6853	1.03 (0.94–1.12)
	*Use 2–3.9 years*	641	6315	1.01 (0.93–1.10)	164	1586	1.06 (0.89–1.26)	477	4729	0.99 (0.90–1.10)
	*Use* ≥ *4 years*	1708	18,006	0.94 (0.88–0.99)	351	3382	1.05 (0.93–1.19)	1357	14,624	0.91 (0.85–0.98)
	*P for trend*	0.088			0.443			0.020		
*Molecular subtype*										
ER +	*Never use*	13,089	130,658	1 (ref.)	6889	68,695	1 (ref.)	6200	61,963	1 (ref.)
	*Past use*	1340	12,828	0.99 (0.93–1.06)	309	3286	0.92 (0.81–1.04)	1031	9542	1.01 (0.94–1.09)
	*Use* < *2 years*	797	7917	0.99 (0.92–1.07)	211	2291	0.93 (0.80–1.08)	586	5626	1.02 (0.93–1.12)
	*Use 2–3.9 years*	498	5191	0.95 (0.86–1.05)	130	1353	0.99 (0.81–1.19)	368	3838	0.94 (0.84–1.06)
	*Use* ≥ *4 years*	1422	14,866	0.94 (0.88–1.01)	312	2885	1.09 (0.96–1.24)	1110	11,981	0.91 (0.85–0.98)
	*P for trend*	0.065			0.514			0.016		
ER-	*Never use*	1928	19,597	1 (ref.)	1059	10,690	1 (ref.)	869	8907	1 (ref.)
	*Past use*	208	1918	1.06 (0.90–1.25)	48	457	1.05 (0.77–1.44)	160	1461	1.06 (0.87–1.28)
	*Use* < *2 years*	135	1212	1.13 (0.92–1.37)	44	341	1.27 (0.90–1.80)	91	871	1.07 (0.84–1.36)
	*Use 2–3.9 years*	94	810	1.17 (0.93–1.48)	27	182	1.45 (0.94–2.25)	67	628	1.08 (0.82–1.42)
	*Use* ≥ *4 years*	213	2243	0.96 (0.81–1.13)	30	410	0.73 (0.49–1.09)	183	1833	1.01 (0.83–1.22)
	*P for trend*	0.913			0.831			0.823		
Luminal A	*Never use*	4640	45,855	1 (ref.)	2480	24,578	1 (ref.)	2160	21,277	1 (ref.)
	*Past use*	439	4275	0.96 (0.86–1.07)	112	1211	0.89 (0.72–1.10)	327	3064	0.99 (0.87–1.13)
	*Use* < *2 years*	229	2684	0.84 (0.73–0.97)	69	837	0.83 (0.64–1.08)	160	1847	0.85 (0.72–1.01)
	*Use 2–3.9 years*	164	1714	0.95 (0.80–1.13)	50	496	1.02 (0.75–1.39)	114	1218	0.93 (0.76–1.14)
	*Use* ≥ *4 years*	464	4832	0.94 (0.84–1.06)	108	1068	1.02 (0.81–1.28)	356	3764	0.93 (0.82–1.06)
	*P for trend*	0.162			0.808			0.167		
Luminal B HER2-	*Never use*	6342	63,876	1 (ref.)	3322	33,371	1 (ref.)	3020	30,505	1 (ref.)
	*Past use*	675	6224	1.04 (0.95–1.14)	151	1550	0.95 (0.79–1.13)	524	4674	1.06 (0.95–1.18)
	*Use* < *2 years*	413	3839	1.06 (0.95–1.19)	107	1056	1.03 (0.83–1.27)	306	2783	1.08 (0.95–1.23)
	*Use 2–3.9 years*	252	2614	0.95 (0.83–1.10)	65	641	1.04 (0.79–1.36)	187	1973	0.93 (0.79–1.09)
	*Use* ≥ *4 years*	711	7377	0.95 (0.87–1.04)	154	1372	1.12 (0.93–1.35)	557	6005	0.91 (0.82–1.01)
	*P for trend*	0.308			0.278			0.087		
Luminal B HER2 +	*Never use*	1301	12,935	1 (ref.)	736	7356	1 (ref.)	565	5579	1 (ref.)
	*Past use*	116	1151	0.97 (0.79–1.21)	33	347	0.73 (0.55–0.98)	83	804	0.95 (0.73–1.24)
	*Use* < *2 years*	83	750	1.09 (0.85–1.39)	25	256	1.02 (0.66–1.59)	58	494	1.12 (0.82–1.51)
	*Use 2–3.9 years*	44	472	0.93 (0.67–1.30)	10	146	0.77 (0.39–1.49)	34	326	1.00 (0.68–1.47)
	*Use* ≥ *4 years*	120	1332	0.89 (0.71–1.11)	34	275	1.32 (0.87–1.99)	86	1057	0.76 (0.59–0.99)
	*P for trend*	0.373			0.435			0.103		
HER2 +	*Never use*	618	6115	1 (ref.)	366	3640	1 (ref.)	252	2475	1 (ref.)
	*Past use*	48	567	0.82 (0.59–1.13)	10	162	0.64 (0.33–1.25)	38	405	1.05 (0.62–1.77)
	*Use* < *2 years*	41	359	1.17 (0.82–1.68)	15	110	1.44 (0.79–2.62)	26	249	1.08 (0.68–1.69)
	*Use 2–3.9 years*	26	237	1.11 (0.71–1.72)	7	63	1.14 (0.50–2.61)	19	174	1.05 (0.62–1.77)
	*Use* ≥ *4 years*	58	632	0.95 (0.69–1.31)	12	125	0.97 (0.50–1.90)	46	507	0.90 (0.62–1.31)
	*P for trend*	0.942			0.806			0.750		
TNBC	*Never use*	1139	11,688	1 (ref.)	609	6178	1 (ref.)	530	5510	1 (ref.)
	*Past use*	142	1191	1.17 (0.96–1.44)	34	257	1.32 (0.90–1.93)	108	934	1.13 (0.89–1.43)
	*Use* < *2 years*	84	748	1.11 (0.87–1.43)	25	204	1.20 (0.76–1.89)	59	544	1.08 (0.80–1.46)
	*Use 2–3.9 years*	58	490	1.17 (0.87–1.57)	17	106	1.58 (0.90–2.77)	41	384	1.04 (0.73–1.48)
	*Use* ≥ *4 years*	129	1403	0.91 (0.73–1.13)	16	265	0.60 (0.35–1.04)	113	1138	0.98 (0.77–1.25)
	*P for trend*	0.683			0.568			0.886		

*Abbreviations* Hazard ratio (HR), Confidence interval (CI), Oestrogen receptor (ER), Human epidermal growth factor receptor 2 (HER2), Triple-negative breast cancer (TNBC)

^a^Adjusted for age in years, education, income quartiles, marital status, country of origin, number of children, and ever use of other anti-platelets, beta-blockers, angiotensin-converting enzyme inhibitors, angiotensin receptor blockers, calcium channel blockers, diuretics, statins, antidiabetics, non-steroidal anti-inflammatory drugs, and menopausal hormone therapy

**Table 4:** Association between use of low-dose aspirin and incidence of breast cancer in Norway 2004–2018, by molecular subtype, stage, and age

	Low-dose aspirin	Cases	Person-years	HR^a^ (95% CI)	Age (years)					
					50–64.9			≥ 65		
					Cases	Person-years	HR^a^ (95% CI)	Cases	Person-years	HR^a^ (95% CI)
ER + Local	*Never use*	11,825	8,670,359	1 (ref.)	7575	5,543,101	1 (ref.)	4250	3,127,258	1 (ref.)
	*Current use*	1876	1,497,420	0.96 (0.91–1.02)	566	402,125	0.95 (0.86–1.05)	1310	1,095,295	0.97 (0.90–1.04)
	*Past use*	824	586,836	1.02 (0.95–1.10)	248	161,296	0.97 (0.85–1.11)	576	425,541	1.04 (0.95–1.14)
Regional	*Never use*	5259	8,670,359	1 (ref.)	3387	5,543,101	1 (ref.)	1872	3,127,258	1 (ref.)
	*Current use*	937	1,497,420	0.97 (0.89–1.06)	273	402,125	1.09 (0.94–1.26)	664	1,095,295	0.93 (0.84–1.03)
	*Past use*	374	586,836	0.94 (0.84–1.06)	85	161,296	0.80 (0.64–1.00)	289	425,541	0.99 (0.87–1.13)
Distant	*Never use*	597	8,670,359	1 (ref.)	322	5,543,101	1 (ref.)	275	3,127,258	1 (ref.)
	*Current use*	120	1,497,420	1.04 (0.82–1.31)	22	402,125	0.95 (0.58–1.56)	98	1,095,295	1.07 (0.82–1.39)
	*Past use*	53	586,836	1.13 (0.83–1.53)	8	161,296	0.85 (0.41–1.75)	45	425,541	1.20 (0.86–1.68)
ER-Local	*Never use*	1519	8,670,359	1 (ref.)	1000	5,543,101	1 (ref.)	519	3,127,258	1 (ref.)
	*Current use*	268	1,497,420	1.07 (0.92–1.25)	85	402,125	1.12 (0.86–1.46)	183	1,095,295	1.04 (0.85–1.26)
	*Past use*	108	586,836	1.07 (0.87–1.32)	32	161,296	1.02 (0.71–1.47)	76	425,541	1.06 (0.82–1.38)
Regional	*Never use*	1001	8,670,359	1 (ref.)	636	5,543,101	1 (ref.)	365	3,127,258	1 (ref.)
	*Current use*	171	1,497,420	0.93 (0.77–1.13)	41	402,125	0.91 (0.63–1.31)	130	1,095,295	0.93 (0.74–1.17)
	*Past use*	81	586,836	1.08 (0.84–1.38)	21	161,296	1.11 (0.71–1.75)	60	425,541	1.05 (0.79–1.42)
Distant	*Never use*	144	8,670,359	1 (ref.)	80	5,543,101	1 (ref.)	64	3,127,258	1 (ref.)
	*Current use*	31	1,497,420	1.01 (0.64–1.60)	6	402,125	0.89 (0.34–2.33)	25	1,095,295	1.06 (0.63–1.80)
	*Past use*	9	586,836	0.80 (0.39–1.62)	1	161,296	-	8	425,541	0.94 (0.43–2.03)

*Abbreviations* Hazard ratio (HR), Confidence interval (CI), Oestrogen receptor (ER)

^a^ Adjusted for age in years (the underlying time scale), education, income quartiles, marital status, country of origin, number of children, and ever use of other anti-platelets, beta-blockers, angiotensin-converting enzyme inhibitors, angiotensin receptor blockers, calcium channel blockers, diuretics, statins, antidiabetics, non-steroidal anti-inflammatory drugs, and menopausal hormone therapy

In women with a BMI ≥ 25, we found an association between current use of low-dose aspirin, as compared to never use, and reduced risk of ER + BC (HR = 0.91, 95% CI: 0.83–0.99), but not in women with a BMI < 25 (HR = 1.00, 95% CI: 0.88–1.12) (Table 5). For ER + BC, in women with a BMI ≥ 25, HRs for past use and current use of < 2, 2–3.9 and ≥ 4 years were 0.95 (95% CI: 0.83–1.07), 0.96 (95% CI: 0.82–1.12), 0.96 (95% CI: 0.79–1.15) and 0.86 (95% CI: 0.75–0.97), respectively (*p*-value for trend = 0.024) (Table 6).

**Table 5:** Association between use of low-dose aspirin and incidence of breast cancer in Norway 2004–2018, by molecular subtype and body mass index (*n* = 450,080)

		BMI (kg/ m^2^)					
		< 25			≥ 25		
	Low-dose aspirin	Cases	Person-years	HR^a^ (95% CI)	Cases	Person-years	HR^a^ (95% CI)
	*Never use*	4858	2,185,363	1 (ref.)	5469	2,167,082	1 (ref.)
	*Current use*	508	200,936	1.03 (0.93–1.15)	916	341,753	0.91 (0.84–0.99)
	*Past use*	259	93,207	1.06 (0.93–1.21)	410	128,457	1.04 (0.94–1.16)
*Molecular subtype*							
ER +	*Never use*	4090	2,185,363	1 (ref.)	4662	2,167,082	1 (ref.)
	*Current use*	415	200,936	1.00 (0.88–1.12)	771	341,753	0.91 (0.83–0.99)
	*Past use*	208	93,207	0.98 (0.85–1.14)	335	128,457	1.00 (0.89–1.12)
ER-	*Never use*	588	2,185,363	1 (ref.)	615	2,167,082	1 (ref.)
	*Current use*	64	200,936	1.12 (0.83–1.51)	115	341,753	0.97 (0.77–1.23)
	*Past use*	50	93,207	1.80 (1.32–2.46)	55	128,457	1.20 (0.90–1.60)
Luminal A	*Never use*	1616	2,185,363	1 (ref.)	1661	2,167,082	1 (ref.)
	*Current use*	172	200,936	0.99 (0.83–1.20)	264	341,753	0.86 (0.74–0.99)
	*Past use*	71	93,207	0.82 (0.64–1.05)	125	128,457	1.03 (0.85–1.24)
Luminal B HER2-	*Never use*	1860	2,185,363	1 (ref.)	2297	2,167,082	1 (ref.)
	*Current use*	194	200,936	1.01 (0.85–1.20)	381	341,753	0.93 (0.81–1.05)
	*Past use*	103	93,207	1.09 (0.88–1.34)	169	128,457	1.04 (0.88–1.23)
Luminal B HER2 +	*Never use*	394	2,185,363	1 (ref.)	463	2,167,082	1 (ref.)
	*Current use*	33	200,936	1.10 (0.73–1.65)	72	341,753	0.86 (0.64–1.15)
	*Past use*	20	93,207	1.25 (0.78–2.00)	27	128,457	0.86 (0.57–1.28)
HER2 +	*Never use*	209	2,185,363	1 (ref.)	199	2,167,082	1 (ref.)
	*Current use*	23	200,936	1.26 (0.76–2.11)	33	341,753	0.90 (0.58–1.39)
	*Past use*	11	93,207	1.23 (0.65–2.33)	10	128,457	0.70 (0.36–1.35)
TNBC	*Never use*	322	2,185,363	1 (ref.)	361	2,167,082	1 (ref.)
	*Current use*	31	200,936	0.90 (0.59–1.38)	76	341,753	1.06 (0.79–1.42)
	*Past use*	35	93,207	2.10 (1.43–3.08)	39	128,457	1.42 (0.99–2.02)

*Abbreviations* Body mass index (BMI), Hazard ratio (HR), Confidence interval (CI), Oestrogen receptor (ER), Human epidermal growth factor receptor 2 (HER2), Triple-negative breast cancer (TNBC)

^a^Adjusted for age in years (the underlying time scale), education, income quartiles, marital status, country of origin, number of children, and ever use of other anti-platelets, beta-blockers, angiotensin-converting enzyme inhibitors, angiotensin receptor blockers, calcium channel blockers, diuretics, statins, antidiabetics, non-steroidal anti-inflammatory drugs, and menopausal hormone therapy

**Table 6:** Association between duration of low-dose aspirin use and incidence of breast cancer in Norway 2004–2018, by molecular subtype and body mass index

	Low-dose aspirin	BMI (kg/ m^2^)					
		< 25			≥ 25		
		Cases	Controls	HR^a^ (95% CI)	Cases	Controls	HR^a^ (95% CI)
Total population	*Never use*	3844	38,920	1 (ref.)	4379	43,059	1 (ref.)
	*Past use*	245	2364	1.03 (0.90–1.19)	387	3828	0.98 (0.88–1.10)
	*Use* < *2 years*	160	1444	1.16 (0.98–1.39)	244	2509	0.96 (0.83–1.11)
	*Use 2–3.9 years*	92	971	1.01 (0.80–1.26)	168	1671	1.00 (0.84–1.18)
	*Use* ≥ *4 years*	211	2221	1.00 (0.85–1.18)	410	4810	0.84 (0.75–0.95)
	*P for trend*	0.652			0.010		
*Molecular subtype*							
ER +	*Never use*	3305	32,874	1 (ref.)	3776	36,974	1 (ref.)
	*Past use*	194	1997	0.96 (0.82–1.12)	318	3270	0.95 (0.83–1.07)
	*Use* < *2 years*	132	1248	1.11 (0.92–1.35)	204	2110	0.96 (0.82–1.12)
	*Use 2–3.9 years*	79	820	1.02 (0.80–1.31)	137	1426	0.96 (0.79–1.15)
	*Use* ≥ *4 years*	173	1891	0.96 (0.81–1.15)	357	4140	0.86 (0.75–0.97)
	*P for trend*	0.938			0.024		
ER-	*Never use*	476	4966	1 (ref.)	507	5128	1 (ref.)
	*Past use*	47	294	1.65 (1.17–2.34)	52	465	1.10 (0.80–1.51)
	*Use* < *2 years*	23	159	1.55 (0.96–2.50)	36	331	0.98 (0.66–1.44)
	*Use 2–3.9 years*	9	128	0.78 (0.38–1.60)	26	205	1.18 (0.75–1.84)
	*Use* ≥ *4 years*	27	273	1.10 (0.69–1.73)	46	551	0.76 (0.53–1.08)
	*P for trend*	0.525			0.291		

*Abbreviations* Body mass index (BMI), Hazard ratio (HR), Confidence interval (CI), Oestrogen receptor (ER)

^a^Adjusted for age in years, education, income quartiles, marital status, country of origin, number of children, and ever use of other anti-platelets, beta-blockers, angiotensin-converting enzyme inhibitors, angiotensin receptor blockers, calcium channel blockers, diuretics, statins, antidiabetics, non-steroidal anti-inflammatory drugs, and menopausal hormone therapy

In the analysis where symptomatic BC was the event of interest, HR for the association between current use of low-dose aspirin, as compared to never use, and risk of ER + BC among all women was estimated at 0.98 (95% CI: 0.93–1.03) and the corresponding estimate among women aged ≥ 65 years was 0.96 (95% CI: 0.91–1.02). The corresponding estimates for the analysis where screen-detected ER + BC was the event of interest were 0.92 (95% CI: 0.84–1.00) for all women and 0.87 (95% CI: 0.76–0.99) for women aged ≥ 65, respectively. In the analyses where women were censored at the time of their first filled prescription of HT, the HR for the association between current use of low-dose aspirin, as compared to never use, and risk of ER + BC among all women was estimated at 0.94 (95% CI: 0.90–1.00), and the corresponding estimate among women aged ≥ 65 years was 0.94 (95% CI: 0.87–1.01). When applying a 2-month grace period, the HR for the association between current use of low-dose aspirin, as compared to never use, and risk of ER + BC among all women was estimated at 0.95 (95% CI: 0.90–0.99) and at 0.95 (95% CI: 0.90–1.00) among women aged ≥ 65 years. The corresponding estimates when applying a 6-month grace period were 0.96 (95% CI: 0.92–1.00) and 0.95 (95% CI: 0.90–1.00), respectively.

## Discussion

To the best of our knowledge, this is the largest study assessing the association between use of low-dose aspirin and risk of BC. Most of the previous studies reported no association [3], possibly because they included smaller study populations, or because they did not stratify by women’s characteristics or cancer molecular subtypes. Our large study population, in combination with the comprehensive linkage of pharmaceutical, patient, and clinical information, allowed us to study the association in detail in various subgroups defined by women’s age and BMI and cancer molecular subtype. We found that current use of low-dose aspirin was associated with a slightly reduced risk of ER + BC in women aged ≥ 65 years and women with a BMI ≥ 25. The associations became more pronounced with longer durations of low-dose aspirin use. The evidence of an association was particularly clear for the luminal A BC. No association was found with ER- BC. Prostaglandins are synthesised by the COX enzyme, which is inhibited by aspirin [4]. Prostaglandins are involved in different processes of cancer development, such as suppression of apoptosis and promotion of cell proliferation, invasiveness, and migration. It has also been suggested that prostaglandins might increase the activity of aromatase [5, 6], an enzyme involved in the conversion of androstenedione to estrone, the major oestrogen in postmenopausal women. Therefore, higher prostaglandin levels could result in an increased risk of BC, in particular ER + BC. Hence, reducing the prostaglandin levels by inhibiting the COX enzymes with aspirin might result in a lower risk of BC. Peripheral aromatase expression in breast adipose tissue is responsible for most of the oestrogen production in postmenopausal women, and the aromatase activity in breast adipose tissue is increasing with age, also after menopause [21]. Hence, the regulation of aromatase activity in the breast might be particularly important for the development of ER + BC among older women. Therefore, it is plausible that older women might benefit more from the suggested decreased aromatase activity compared to younger women. In line with this hypothesis, we found an association between low-dose aspirin use and reduced risk of ER + BC in older women (aged ≥ 65 years). Consistent with our results of an association only among women aged ≥ 65 years, Hurwitz et al. analysed a cohort of 423,495 women among whom 9730 BC cases occurred [22], and reported an indication of an association between aspirin use (including both low-dose and regular-dose) and risk of any type of BC among women aged ≥ 70 years (HR = 0.86, 95% CI: 0.74–1.01), but not among women aged 50–59 years (HR = 0.95, 95% CI: 0.86–1.05), or 60–69 years (HR = 0.98, 95% CI: 0.92–1.04).

In line with our findings, Ma et al. performed a meta-analysis in 2020 [3], including 14 observational studies that assessed the association between aspirin use and BC risk separately by ER status, and reported that the use of aspirin (including both low-dose and regular-dose) was associated with a reduced risk of ER + BC (relative risk [RR]: 0.89, 95% CI: 0.82–0.97), but not ER- BC (RR = 0.96, 95% CI: 0.84–1.09). The fact that this association only held for ER + BC is compatible with the hypothesis that aspirin exerts an anti-cancer effect by lowering oestrogen levels [5, 6]. In contrast to our study, a Danish cohort study and a Spanish case–control study failed to find an association with ER + BC [23, 24], possibly because they included smaller study populations and larger proportions of young women compared to our study. The Danish study included 28,965 women aged 50–65 years, and the Spanish study included 1736 cases (mean age: 56.4 years) and 1909 controls (mean age: 59.0 years).

Inflammation can cause DNA damage resulting in changes that lead to cancer development and progression [25]. Reducing the levels of inflammatory mediators, such as prostaglandins, by inhibiting the COX enzymes, may thus reduce the risk of cancer [4]. Overweight and obesity are conditions with increased levels of inflammatory mediators, such as prostaglandins [26]. Therefore, women with a higher BMI might benefit more from the use of aspirin compared to women with a lower BMI. Our results of an association between low-dose aspirin use and a reduced risk of BC among women with a BMI ≥ 25, but not among women with a BMI < 25, is in line with this hypothesis. Only a few studies have investigated the association between aspirin use and BC risk by BMI. In line with our results, Cui et al. (2674 BC cases and 2361 controls) reported that regular use of low-dose aspirin was associated with a reduced risk of any type of BC among women with a BMI ≥ 25 (odds ratio [OR] = 0.74, 95% CI: 0.59–0.94) [27], but not among those with a BMI < 25 (OR = 0.99, 95% CI: 0.73–1.34). Hurwitz et al. reported an indication of an association between aspirin use (including both low-dose and regular-dose) and risk of any type of BC among women with a BMI ≥ 30 (HR = 0.93, 95% CI: 0.84–1.02) [22], but not among those with a BMI < 30. Other studies have reported no evidence in support of an association between aspirin use and BC risk at any BMI level [24, 28].

This study’s main strength is its large study population of more than 1 million women, which allowed us to analyse the association of interest in different subgroups according to the population’s characteristics. Another strength of this study is its population-based approach, which minimised the risk of selection bias. The linkage with the Norwegian Prescription Database ensured detailed information on low-dose aspirin use without risk of recall bias. The linkage between population-based registries also facilitated the inclusion of high-quality information on several relevant confounders such as income, education, and the number of children. However, this study has several limitations. We do not have information on comorbid conditions. To address this limitation, we adjusted for concomitant use of cardiovascular medications, statins, antidiabetics, NSAIDs, and HT as a proxy for health conditions. Due to insufficient data, we did not adjust for lifestyle risk factors, such as physical activity and alcohol consumption, which might be potential confounders of our association of interest. If increased alcohol consumption and lack of physical activity were associated with a higher likelihood of using low-dose aspirin and a higher risk of BC, then not adjusting for these variables would bias the results in the direction of a detrimental effect of low-dose aspirin (i.e., higher HR and underestimation of the protective effect of low-dose aspirin). We tried to overcome this by adjusting for medications used to treat comorbid conditions that might depend on lifestyle factors such as alcohol consumption and physical activity. The Norwegian Prescription Database contains accurate information on filled prescriptions but contains no information on actual use of the medications, adherence, or on actual duration of use. We also did not have access to information on aspirin in regular doses. This makes it difficult to compare our results to studies that included regular-dose aspirin. It might also have biased the results: if, for example, regular-dose aspirin has a protective effect against BC and it was prescribed more to low-dose aspirin non-users than users, then we would have underestimated the association between low-dose aspirin and BC (i.e., biased towards a HR of 1). However, it should not be a concern because regular-dose aspirin is rarely used in Norway [29]: in 2020, only 0.2/1,000 inhabitants (in the overall Norwegian population) were dispensed regular-dose aspirin in Norway. Our data do not contain information on BMI from the date when the follow-up began; instead, we used the BMI measurement closest in time to the start of follow-up, with a maximum of 5 years before or after the start of follow-up. However, the estimates for the association between low-dose aspirin and BC risk did not vary substantially when reducing the length of this interval (data not shown). Furthermore, it is important to highlight that women with a BMI measurement might not be representative of the whole population, as they were more educated and had a higher income compared to the whole population. This might reduce the generalisability of our results. We could not assess the influence of the dose of aspirin (i.e., mg of the filled pills), because the vast majority of the filled prescriptions of low-dose aspirin was 75 mg. However, in Ma’s meta-analysis, the aspirin dose seems to be of small or no importance [3]. A final limitation is potential selection bias in the nested case–control study, rising from selecting women with ≥ 4 years of follow-up. However, the estimates for the association between low-dose aspirin use and risk of BC were similar when not selecting women based on follow-up time in the nested case–control study, and in the cohort study (data not shown), indication that selection bias is not of substantial concern.

## Conclusion

Use of low-dose aspirin was associated with a small reduction in the risk of ER + BC in women aged ≥ 65 years and overweight women.

## Supplementary Information

Below is the link to the electronic supplementary material.

Supplementary file1 (DOCX 58 kb)

## Data Availability

Due to Norwegian law, we are not allowed to make the data publicly available. However, the data can be requested from the registry holders.

## Declarations

### Disclaimar

The Norwegian Institute of Public Health is not responsible for the content of publications, analyses or conclusions based on the provided data. The interpretation and reporting of these data are the sole responsibility of the authors, and no endorsement by the Cancer Registry of Norway is intended nor should be inferred.
