# Supplementary material for: Low-dose aspirin and risk of breast cancer: a Norwegian population-based cohort study of one million women
Source: Eur J Epidemiol. 2023 Mar 6;38(4):413–26. doi: 10.1007/s10654-023-00976-8 (PMC10082109; doi:10.1007/s10654-023-00976-8)
Supplement: Supplementary file 1 — Supplementary file1 (DOCX 58 kb) [file 10654_2023_976_MOESM1_ESM.docx]

**Supplementary material**


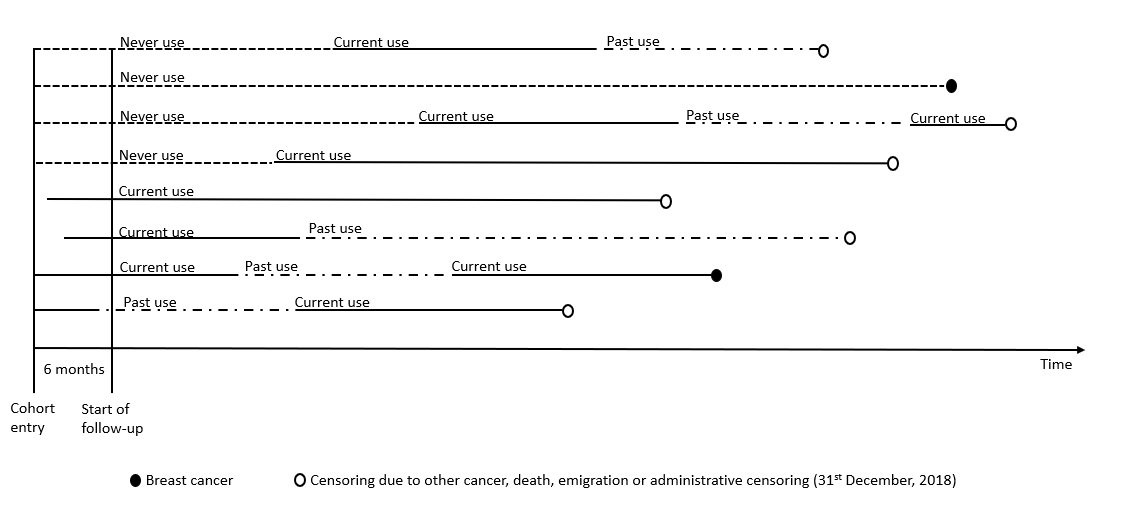


**Supplementary Figure 1** Follow-up definition of study participants

| **Supplementary Table 1** Medications and corresponding Anatomical Therapeutic Chemical (ATC) codes | |
| --- | --- |
| **Medications** | **ATC code** |
| Low-dose aspirin | B01AC06, B01AC56 |
| Other anti-platelets | B01AC01-05, B01AC7-30 |
| β-blockers | C07 |
| Angiotensin-converting enzyme inhibitors | C09A, C09B |
| Angiotensin receptor blockers | C09C, C09D |
| Calcium channel blockers | C08, C09BB, C09DB |
| Diuretics | C03, C07B, C09BA, C09DA |
| Statins | C10AA, C10BA02, C10BA05, C10BA06 |
| Antidiabetics | A10 |
| Non-steroidal anti-inflammatory drugs | M01A |
| Menopausal hormone therapy | G03C, G03F |

| **Supplementary Table 2** Baseline characteristics in the different study populations used for the different analyses, Norway 2004-2018 | | | |
| --- | --- | --- | --- |
|  | Full cohort (N=1,083,629) | BMI cohort (N=450,080) | NCC population ^a^ (N=196,799) |
| **Age (years) at the start of follow-up** |  |  |  |
| Median (Q1, Q3) | 52.7 (50.0, 62.7) | 50.9 (50.0, 57.6) | 57.0 (50.9, 64.4) |
| **Highest education level** |  |  |  |
| None/primary school | 310,357 (28.6%) | 101,973 (22.7%) | 57,790 (29.4%) |
| Secondary school | 464,285 (42.8%) | 213,198 (47.4%) | 90,085 (45.8%) |
| University | 274,830 (25.4%) | 132,412 (29.4%) | 44,036 (22.4%) |
| Missing | 34,157 (3.2%) | 2,497 (0.6%) | 4,888 (2.5%) |
| **Income (Norwegian kroner)** |  |  |  |
| Q1 (<154,000) | 266,058 (24.6%) | 71,949 (16.0%) | 56,499 (28.7%) |
| Q2 (154,000–258,000) | 266,057 (24.6%) | 114,359 (25.4%) | 57,878 (29.4%) |
| Q3 (258,001–385,000) | 266,057 (24.6%) | 147,398 (32.7%) | 51,584 (26.2%) |
| Q4 (>385,000) | 266,057 (24.6%) | 116,172 (25.8%) | 27,290 (13.9%) |
| Missing | 19,400 (1.8%) | 202 (0.0%) | 3,548 (1.8%) |
| **Marital status** |  |  |  |
| Married/partnered | 418,097 (38.6%) | 143,118 (31.8%) | 68,757 (34.9%) |
| Not married/partnered | 640,675 (59.1%) | 305,171 (67.8%) | 123,793 (62.9%) |
| Missing | 24,857 (2.3%) | 1,791 (0.4%) | 4,249 (2.2%) |
| **Country of origin** |  |  |  |
| Norway | 950,796 (87.7%) | 408,188 (90.7%) | 179,429 (91.2%) |
| Other Nordic countries ^b^ | 34,571 (3.2%) | 13,611 (3.0%) | 5,420 (2.8%) |
| Rest of the world | 98,262 (9.1%) | 28,281 (6.3%) | 11,950 (6.1%) |
| **Children** |  |  |  |
| 0 | 139,859 (12.9%) | 40,345 (9.0%) | 22,481 (11.4%) |
| 1 | 142,211 (13.1%) | 54,129 (12.0%) | 24,305 (12.4%) |
| 2 | 408,880 (37.7%) | 191,364 (42.5%) | 75,823 (38.5%) |
| ≥3 | 392,679 (36.2%) | 164,242 (36.5%) | 74,190 (37.7%) |
| **BMI (kg/ m^2^)** |  |  |  |
| <25 | 217,882 (20.1%) | 217,882 (48.4%) | 46,333 (23.5%) |
| ≥25 | 232,198 (21.4%) | 232,198 (51.6%) | 50,105 (25.5%) |
| Missing | 633,549 (58.5%) | - | 100,361 (51.0%) |
| **Ever use of drugs** |  |  |  |
| Low-dose aspirin | 257,442 (23.8%) | 94,518 (21.0%) | 59,449 (30.2%) |
| Other anti-platelets | 70,911 (6.5%) | 24,291 (5.4%) | 16,721 (8.5%) |
| Beta-blockers | 271,931 (25.1%) | 100,711 (22.4%) | 61,150 (31.1%) |
| Calcium channel blockers | 216,6711 (20.0%) | 81,279 (18.1%) | 49,565 (25.2%) |
| Angiotensin-converting enzyme inhibitors | 130,627 (12.1%) | 45,446 (10.1%) | 29,260 (14.9%) |
| Angiotensin receptor blockers | 280,389 (25.9%) | 118,027 (26.2%) | 62,862 (31.9%) |
| Diuretics | 331,431 (30.6%) | 121,763 (27.1%) | 73,890 (37.5%) |
| Statins | 343,267 (31.7%) | 147,759 (32.8%) | 78,340 (39.8%) |
| Antidiabetics | 86,617 (7.9%) | 31,215 (6.9%) | 17,726 (9.0%) |
| Non-steroidal anti-inflammatory drugs | 770,446 (71.1%) | 365,770 (81.3%) | 156,405 (79.5%) |
| Menopausal hormone therapy | 418,224 (37.6%) | 226,594 (50.3%) | 91,419 (46.5%) |
| Abbreviations: Quartile (Q), Body mass index (BMI), Nested case-control (NCC) | | |  |
| ^a^ Some individuals in the NCC study were included both as a case and as a control, in this table they are included only once  ^b^ Includes Denmark, Finland, Iceland, and Sweden | | | |

| **Supplementary Table 3** Characteristics at index for breast cancer cases and matched controls, Norway 2004-2018 | | |
| --- | --- | --- |
|  | Controls (N=205,230) | BC cases (N=20,523) |
| **Age (years) at index** |  |  |
| Median (Q1, Q3) | 66.0 (60.3, 73.6) | 66.0 (60.3, 73.6) |
| **Highest education level** |  |  |
| None/primary school only | 59,841 (29.2%) | 5,301 (25.8%) |
| Secondary school | 93,629 (45.6%) | 9,982 (48.6%) |
| University | 45,669 (22.3%) | 5,081 (24.8%) |
| Missing | 6,091 (3.0%) | 159 (0.8%) |
| **Income (Norwegian kroner)** |  |  |
| Q1 (<154,000) | 50,194 (24.5%) | 4,538 (22.1%) |
| Q2 (154,000–258,000) | 49,933 (24.3%) | 4,799 (23.4%) |
| Q3 (258,001–385,000) | 49,609 (24.2%) | 5,123 (25.0%) |
| Q4 (>385,000) | 49,207 (24.0%) | 5,525 (26.9%) |
| Missing | 6,287 (3.1%) | 538 (2.6%) |
| **Marital status** |  |  |
| Married/partnered | 87,696 (42.7%) | 9,077 (44.2%) |
| Not married/partnered | 117,534 (57.3%) | 11,446 (55.8%) |
| **Country of origin** |  |  |
| Norway | 187,179 (91.2%) | 18,861 (91.9%) |
| Other Nordic countries^a^ | 5,627 (2.7%) | 555 (2.7%) |
| Rest of the world | 12,424 (6.1%) | 1,107 (5.4%) |
| **Children** |  |  |
| 0 | 23,479 (11.4%) | 2,239 (10.9%) |
| 1 | 25,013 (12.2%) | 2,901 (14.1%) |
| 2 | 78,708 (38.4%) | 8,317 (40.5%) |
| ≥3 | 78,030 (38.0%) | 7,066 (34.4%) |
| **BMI (kg/ m^2^)** |  |  |
| <25 | 45,920 (22.4%) | 4,552 (22.2%) |
| ≥25 | 55,877 (27.2%) | 5,588 (27.3%) |
| Missing | 103,433 (50.4%) | 10,383 (50.5%) |
|  |  |  |
| **Ever use of other drugs** |  |  |
| Low-dose aspirin | 49,364 (24.1%) | 4,953 (24.1%) |
| Other anti-platelets | 11,020 (5.4%) | 1,013 (4.9%) |
| Beta-blockers | 51,106 (24.9%) | 5,344 (26.0%) |
| Calcium channel blockers | 37,460 (18.3%) | 4,002 (19.5%) |
| Angiotensin-converting enzyme inhibitors | 23,110 (11.1%) | 2,344 (11.4%) |
| Angiotensin receptor blockers | 51,569 (25.1%) | 5,596 (27.3%) |
| Diuretics | 64,701 (31.5%) | 7,021 (34.2%) |
| Statins | 66,084 (32.5%) | 6,462 (31.5%) |
| Antidiabetics | 14,566 (7.1%) | 1,505 (7.3%) |
| Non-steroidal anti-inflammatory drugs | 148,903 (72.6%) | 15,505 (75.5%) |
| Menopausal hormone therapy | 81,328 (39.6%) | 9,529 (46.4%) |
| Abbreviations: Quartile (Q), Body mass index (BMI) | | |
| ^a^ Includes Denmark, Finland, Iceland, and Sweden | | |
